# RIP-Chip analysis supports different roles for AGO2 and GW182 proteins in recruiting and processing microRNA targets

**DOI:** 10.1186/s12859-019-2683-y

**Published:** 2019-04-18

**Authors:** Giovanni Perconti, Patrizia Rubino, Flavia Contino, Serena Bivona, Giorgio Bertolazzi, Michele Tumminello, Salvatore Feo, Agata Giallongo, Claudia Coronnello

**Affiliations:** 1 Istituto di Biomedicina ed Immunologia Molecolare (IBIM) CNR, via Ugo la Malfa 153, 90146 Palermo, Italy; 2 Dipartimento di Scienze e Tecnologie Biologiche Chimiche e Farmaceutiche, Università degli Studi di Palermo, 90128 Palermo, Italy; 3 ATEN Center, Università degli Studi di Palermo, 90128 Palermo, Italy; 4 Dipartimento di Scienze Economiche, Aziendali e Statistiche, Università degli Studi di Palermo, 90128 Palermo, Italy; 5 Fondazione Ri.MED, via Bandiera 11, 90133 Palermo, Italy

**Keywords:** microRNA regulatory activity, RIP-Chip data analysis, RISC proteins AGO2 and GW182, microRNA target prediction

## Abstract

**Background:**

MicroRNAs (miRNAs) are small non-coding RNA molecules mediating the translational repression and degradation of target mRNAs in the cell. Mature miRNAs are used as a template by the RNA-induced silencing complex (RISC) to recognize the complementary mRNAs to be regulated. To discern further RISC functions, we analyzed the activities of two RISC proteins, AGO2 and GW182, in the MCF-7 human breast cancer cell line.

**Methods:**

We performed three RIP-Chip experiments using either anti-AGO2 or anti-GW182 antibodies and compiled a data set made up of the miRNA and mRNA expression profiles of three samples for each experiment. Specifically, we analyzed the input sample, the immunoprecipitated fraction and the unbound sample resulting from the RIP experiment. We used the expression profile of the input sample to compute several variables, using formulae capable of integrating the information on miRNA binding sites, both in the 3’UTR and coding regions, with miRNA and mRNA expression level profiles. We compared immunoprecipitated vs unbound samples to determine the enriched or underrepresented genes in the immunoprecipitated fractions, independently for AGO2 and GW182 related samples.

**Results:**

For each of the two proteins, we trained and tested several support vector machine algorithms capable of distinguishing the enriched from the underrepresented genes that were experimentally detected. The most efficient algorithm for distinguishing the enriched genes in AGO2 immunoprecipitated samples was trained by using variables involving the number of binding sites in both the 3’UTR and coding region, integrated with the miRNA expression profile, as expected for miRNA targets. On the other hand, we found that the best variable for distinguishing the enriched genes in the GW182 immunoprecipitated samples was the length of the coding region.

**Conclusions:**

Due to the major role of GW182 in GW/P-bodies, our data suggests that the AGO2-GW182 RISC recruits genes based on miRNA binding sites in the 3’UTR and coding region, but only the longer mRNAs probably remain sequestered in GW/P-bodies, functioning as a repository for translationally silenced RNAs.

**Electronic supplementary material:**

The online version of this article (10.1186/s12859-019-2683-y) contains supplementary material, which is available to authorized users.

## Background

Argonaute (AGO) proteins and the GW182 protein family (also known as TNRC6 proteins) are involved in the cellular process which leads to gene silencing mediated by miRNAs, small endogenous non-coding RNAs that act as post-transcriptional regulators by base pairing to target mRNAs [1, 2]. While miRNAs guide AGOs to target mRNAs, a direct interaction between AGO and GW182 proteins is required for the assembly of ribonucleoprotein complexes, named RISCs, and the recruitment of additional factors involved in gene silencing, which is ultimately achieved through the degradation of target mRNAs or translational repression [3, 4]. Several studies of higher eukaryotes have indicated that, among the AGO proteins, AGO2 is catalytically active and involved in the mRNA cleavage process, whereas AGO1, 3 and 4 are catalytically inactive and mainly involved in translational repression [4, 5]. In the cell cytoplasm, AGOs, together with GW182/TNRC6A and its mammalian paralogs, TNRC6B and TNRC6C, have a role in executing miRNA-mediated repression, either by silencing or decay, but the proteins also contribute to other functions in the nucleus, such as transcription and splicing control [6, 7]. On the other hand, GW182 is a marker of GW/P-bodies, dynamic cytoplasmic structures containing non-translating mRNAs, that have been associated with the cellular response to stress [8] and were first identified because human autoimmune sera recognized them [9, 10]. Work over the past few years has significantly increased our understanding of the biology of GW/P-bodies in higher and lower eukaryotes. It has been shown that these bodies contain proteins involved in diverse post-transcriptional processes, such as mRNA degradation, nonsense-mediated mRNA decay, translational repression, RNA-mediated gene silencing, and may also function as a cytoplasmic domain for RNA storage.

Furthermore, RNA-binding protein immunoprecipitation, coupled with high-throughput methods for expression profiling, such as gene array (RIP-Chip) or sequencing (RIP-Seq), has allowed the systematic identification of RISC-bound miRNAs and their target mRNA sequences in mammalian cells and the dissection of miRNA-mediated post-transcriptional regulatory networks. This approach has been widely applied to the AGO protein family, through the immunoprecipitation of either exogenously introduced tagged-proteins or endogenous proteins and the subsequent analysis of the associated RNAs [10–13]. So far, few reports have described a similar approach for GW182 and its paralogs using specific antibodies [14, 15], and recently, Meister and co-workers reported a novel method, based on affinity purification, for the simultaneous isolation of all AGO-containing complexes [16].

The RIP-based high-throughput method for expression profiling has been widely used to predict miRNA-target interactions in order to develop algorithms useful for identifying potential miRNA targets. Several algorithms predict potential miRNA targets by considering the binding site characteristics of the analyzed miRNA-target pair, for example, the binding site minimum free energy (miRanda [17]), miRNA seed complementarity and conservation (Targetscan [18]), binding site accessibility (PITA [19]). More recent algorithms consider both miRNA and mRNA paired expression profiles to detect functional miRNA-mRNA pairs. As an example, we mention the web-tool MAGIA [20], which combines the prediction results from Targetscan, PITA and miRanda algorithms and adopts different statistical measures of profile correlation and algorithms for expression profile combination. The expression profile of endogenous miRNAs has been shown to be determinant in predicting RISC machinery functional targets, and it is used by ComiR [21] to predict targets of a set of miRNAs. In addition to such collaborative effects, competition effects have a crucial role in miRNA regulatory function, as shown by the evidence of competing exogenous [22] and endogenous [23] effects. In summary, both miRNA and mRNA expression profiles have a crucial role in determining miRNA binding activity.

In order to get additional insight into the diverse cellular functions of RISCs, we performed RIP-Chip experiments using antibodies specific for AGO2 and GW182/TNRC6A. Data from miRNA and mRNA expression profiles were combined, using existing target prediction results, to compute several variables that served to train and test various support vector machine (SVM) algorithms, searching for the more efficient variables for distinguishing enriched genes in the immunoprecipitated samples.

## Methods

### Cell culture

The MCF-7 human breast cancer cell line was purchased from American Type Culture Collection (ATCC, Rockville, MD, USA). Cells were cultured in Dulbecco’s modified Eagle Medium (DMEM) supplemented with 10% heat-inactivated fetal bovine serum (FBS), glutamine (4 mM) and penicillin/streptomycin (100 μg/ml).

### AGO2/GW182 immunoprecipitation

RNA-binding protein immunoprecipitation (RIP) of RISCs was performed using mouse monoclonal anti-AGO2 (clone 1B1-E2H5, RN003M), rabbit anti-GW182 (TNRC6A, RN033P) and the RIP-Assay Kit for microRNA (MBL International Corporation). Briefly, cells (1.5 × 10^7^) were suspended in 0.3 ml of miLysis buffer, supplemented with protease and RNase inhibitors, after incubation on ice for 10 min and one freeze-thaw cycle; the lysate was diluted five times with lysis buffer, and the cytoplasmic fraction was isolated by centrifugation at 12,000×g at 4 °C for 5 min. To eliminate nonspecific binding, the lysate was incubated with protein A/G-agarose beads (SantaCruz) at 4 °C for 1 h. The precleared lysates were then mixed with mouse anti-AGO2 or rabbit anti-GW182 (15 μg of Ab/mg of lysate) armed beads; the use of preimmune mouse IgG isotype (clone 6H3, M076–3, MBL) and rabbit IgG (PM035, MBL) assessed the specificity of the precipitated immunocomplexes. After incubation overnight at 4 °C on a rocking platform, AGO2-IP and GW182-IP beads were washed three times with ice cold wash buffer. Total RNA (i.e., including mRNAs and miRNAs) was extracted from IP fractions following the two-step method described in the RIP-Assay Kit, while total and unbound fractions were processed using TRIzol LS (ThermoFisher Scientific Inc.), according to the manufacturer’s instructions. For GW182_FT3 and GW182 _IN3 samples, total RNA was isolated using the miRNeasy Mini Kit from Qiagen, as described by Turchinovich and Burwinkel [24], obtaining similar results to those achieved with Trizol. In all cases, synthetic miRNA-39 from *C. elegans* (celmiRNA-39) was added as a spike-in control for miRNA isolation efficiency. RNA was quantified by NanoDrop (ThermoFisher Scientific Inc.), and RNA integrity and quality were assessed using the 2100 Agilent Bioanalyzer.

### Immunoblotting

Cytoplasmic proteins from total (IN), immunoprecipitated (IP) and unbound/flow-through (FT) cell lysates were separated on NuPage Novex 4–12% Bis-Tris precast gels (ThermoFisher Scientific Inc.), then transferred onto a PVDF FL membrane (Sigma-Aldrich). Primary antibodies against GW182 or AGO2 proteins (anti-GW182 and anti-AGO2, MBL International Corporation) were revealed with secondary antibodies, either conjugated to IRDye® 800CW (LI-COR) or Alexa Fluor 680, using the Odyssey infrared imaging system (LI-COR Biosciences) according to the manufacturer’s instructions.

### Microarray gene expression analysis

Cyanine-3 (Cy3) or Cyanine-5 (Cy5) labeled cRNA was prepared from 325 ng RNA for IN and FT samples or from 20 ng RNA for IP sample, using the LowInput QuickAmp Labeling Kit (Agilent), according to the manufacturer’s instructions, followed by RNAeasy mini kit column purification (Qiagen). Dye incorporation and cRNA yield were checked with the NanoDrop ND-1000 spectrophotometer. Hybridization and washing were performed using the in situ Hybridization Plus Kit following the manufacturer’s instructions (Agilent protocol: G4140-90050_GeneExpression_TwoColor_ver._6.9.1). Briefly, 1.0 μg of Cy3- or Cy5-labeled cRNA (specific activity > 9.0 pmol Cy/ug cRNA) was fragmented at 60 °C for 30 min in a reaction volume of 55 μl containing 1x fragmentation buffer and 2x blocking agent. On completion of the fragmentation reaction, 55 μl of 2x hybridization buffer was added to the mixture and hybridized to Whole Human Genome Microarray 4x44K v2 (Agilent-G4845A) for 17 h at 65 °C in a rotating hybridization oven. After hybridization, microarrays were washed for 1 min at room temperature with Wash Buffer1 and 1 min with 37 °C with buffer2, then dried immediately. Slides were scanned on the Agilent DNA Microarray Scanner (G2505B) using the two-color scan setting for 4x44k array slides (Scan Area 61 × 21.6 mm, Scan resolution 5 μm, dye channel PMT set to 100%). The scanned images were analyzed by Feature Extraction Software 9.5.1 (Agilent) using default parameters (protocol: GE2-v5_95 and Grid: 026652_D_F_20110325) to obtain background subtracted, dye normalized and spatially detrended processed signal intensities.

### Microarray miRNA expression analysis

Cy3-labeled cRNA was prepared from 100 ng RNA for IN and FT samples or from 20 ng RNA for IP sample, using the miRNA Microarray System with miRNA Complete Labeling and Hyb Kit, according to the manufacturer’s instructions (Agilent protocol: G4170-90011_miRNA_ver_3.1.1). Briefly, Cy3-labeled RNA, in a reaction volume of 45 μl containing 1x blocking agent and 1x Hi-RPM hybridization buffer, was hybridized to Human miRNA Microarray 8x15K (Agilent-G4470C) for 20 h at 55 °C in a rotating hybridization oven. After hybridization, microarrays were washed as above and dried immediately. Slides were scanned using the one-color scan setting for 8x15k array slides (Scan Area 61 × 21.6 mm, Scan resolution 5 μm, dye channel set to green and green PMT set to 100%). The scanned images were analyzed by Feature Extraction Software 9.5.1 (Agilent) using default parameters (protocol: miRNA-v1_95 and Grid: 021827_D_20081121) to obtain background subtracted and spatially detrended processed signal intensities.

### Reverse transcription and real-time PCR analysis

The reverse transcription reaction was performed using the miScript reverse transcription kit (Qiagen), according to the manufacturer’s instructions. Real-time PCR reagents and miScript primers for miRNAs were from Qiagen. Amplification reactions were performed using a StepOne Plus real-time PCR system (Applied Biosystems), according to the manufacturer’s manual; each reaction had three technical replicates, and data are presented as means ± standard deviation.

For normalization purposes, we used an adaptation of the normalization procedure used in [25]. For each sample, we computed which percentage of the total amount of RNA extracted in the IP experiments corresponded to the amount of RNA used in the RT-qPCR. Input RNA was used as the reference RNA. For each IP sample, a normalization factor was computed by dividing the percentage of RNA in the IP sample by the percentage of RNA in the Input sample. After RT-qPCR, for each miRNA, IP results were first compared with the Input RNA, then divided by the respective normalization factor. Differences between IP samples and IgG controls were calculated based on the 2^−ΔΔC(t)^ method.

### Predicted miRNA-mRNA interaction matrix

All the 3’UTR and coding sequences used to predict miRNA binding sites were selected from Ensembl.org. If the database contained more than one sequence for the same Ensembl ID, the longest sequence was selected. We only considered sequences at least 50 bases long. From Ensembl.org we selected 18,552 3’UTR and 19,420 coding sequences, of which 16,363 mRNAs were included in both sets and in the microarray platform used. MiRNA binding sites were predicted using TargetScan [18], PITA [19] and miRanda [17] scripts. We computed two miRNA-mRNA interaction matrices (*BS*), one for 3’UTR and one for the coding regions, which contained the number of binding sites predicted for each miRNA seed on the selected sequences. For both *BS* matrices, we computed the respective density matrices (*dBS*) by dividing the number of predicted binding sites by the length of the considered sequence.

### Data pre-processing and statistical analysis

Microarray data pre-processing consisted of the following pipeline. The Feature Extraction Software already provided background subtracted, dye normalized and spatially detrended processed signal intensities. Intensities were normalized using the quantile normalization technique. First of all, an average linkage cluster analysis was performed in order to check instrumental replicate consistency, and then the average expression profile of instrumental replicates was computed. The obtained expression profiles were used to perform a post-hoc power analysis specific for microarray studies [26], and we obtained an observed power of 0.7, which implied that 70% of truly enriched genes were expected to be discovered.

The pre-processed expression profiles were compared through hierarchical cluster analysis (average linkage), where distance was computed as dist = 1 – correlation. Genes enriched and underrepresented in IP samples were identified using the Significance Analysis of Microarrays (SAM) algorithm [27], implemented by the samr library in BioconductoR. The samr library associates a q-value with each gene, i.e., the lowest False Discovery Rate at which that gene is called significant. It is like the well-known *p*-value, but adapted to multiple-testing situations. A q-value of 5% was set as the threshold for significance in detecting enriched and underrepresented genes. Enriched genes detected by the SAM algorithm were compared with the enriched genes detected by REA [28], an algorithm developed specifically for RIP-Chip enrichment analysis.

The performance of single variables in distinguishing the enriched and the underrepresented genes was evaluated as the area (AUC) under the receiver operating characteristic (ROC) curve, using the *pROC* R library [29] and Wilcoxon signed test p-value. SVM models were trained with linear kernel using the *e1071* R library. The R library *caret* was used to test the SVM trained models with the Leave One Out Cross Validation (trainControl method = “LOOCV”) testing procedure (train method “svmLinear2”).

## Results

### AGO2 and GW182 proteins complexes handle different mRNA content

To gain new insight into the regulatory networks of gene expression involving functionally diverse RISCs in the cell cytoplasm, we used RIP-Chip to identify mRNAs and miRNAs selectively bound to these complexes in the MCF-7 cell line, which is widely used and representative of luminal breast cancer. We selected AGO2 and GW182 antibodies against core RISC proteins since AGO2 is the most abundantly expressed AGO protein in many cell types, including MCF-7 cells [30], and GW182/TNRC6A has been shown to be the major binding partner for AGO2 [31]. We performed three independent RIP experiment, collecting the IN, IP and FT samples.

**Fig. 1:**
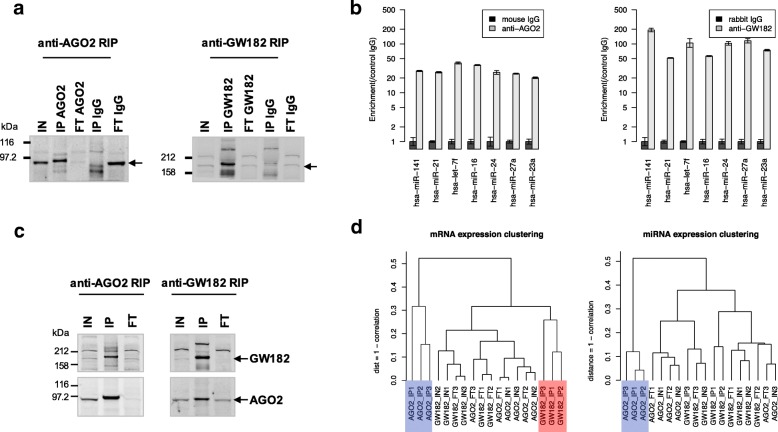
RIP-Chip experiments overview. **a** and **c** Western Blot analysis of proteins immunoprecipitated and co-immunoprecipitated with anti-AGO2 or anti-GW182 antibody (IP). IgGs in a) are the negative controls. IN and FT made up 1% of the cytoplasmic lysate used for each IP sample. GW182 was specifically co-immunoprecipitated with AGO2 (**c**, left panel), and AGO2 was specifically co-immunoprecipitated with GW182 (**c**, right panel). **b** Enrichment analysis of seven highly expressed miRNAs in anti-AGO2 and anti-GW182 IP compared to IgG-IP controls. **d** Average Linkage Cluster analysis of mRNA and miRNA expression profiles of IP, IN and FT samples from three independent experiments; distance is computed as 1- Correlation (Pearson). AGO2-IP and GW182-IP mRNA expression profiles are highlighted in blue and green, respectively. In mRNA expression clustering, we considered all the 16,323 genes with a detected expression level in the samples considered. In miRNA expression clustering, we considered 508 miRNAs with a detected expression level in at least one sample

The efficiency of the AGO2 and GW182 antibodies in IPs was confirmed by the enrichment of both proteins in the IP fractions and their depletion in the FT fractions, while the lack of precipitation of either AGO2 or GW182 protein by control IgG confirmed the specificity of antibodies (Fig. 1a). We also examined, in AGO2-IP and GW182-IP, the enrichment of seven miRNAs highly expressed in the MCF7 cell line [13]. As shown in Fig. 1b, all the analyzed miRNAs were significantly enriched by AGO2 and GW182-IP compared to controls (*p*-value < 0.05, AGO2 or GW182-IP vs IgG-IP). As expected for proteins present in the same complex, Western Blot analysis confirmed the reciprocal co-immunoprecipitation of AGO2 and GW182 (Fig. 1c). Whole genome and miRNA expression profiles, as determined by microarray analysis, gave rise to a novel dataset that is available through the NCBI GEO database (accession ID GSE109667). As shown in Fig. 1d, the cluster analysis performed on whole genome expression profiles revealed that the mRNA expression profiles of the AGO2-IP samples (blue cluster) were homogeneous and different from the GW182-IP mRNA expression profiles (red cluster). The miRNA expression profile clustering showed only one homogenous cluster, the AGO2-IP sample cluster (Fig. 1d, blue cluster). The comparison of AGO2-IP vs IN expression profiles revealed the underrepresentation, in the IP sample, of several miRNAs highly expressed in IN samples, a fact that implies a lower correlation between IP and IN expression profiles (see Additional file 1). On the other hand, GW182-IP and IN miRNA expression profiles were more similar to each other, and such behavior explains the absence of a GW-IP cluster in miRNA expression profile clustering.

We also characterized the two proteins’ behavior by detecting the enriched genes in AGO2-IP and GW182-IP. We observed that the most efficient comparison in retrieving miRNA targets was the one between IP vs FT, with respect to IP vs IN samples. Indeed, GSEA analysis (see Additional file 2) showed more miRNA predicted targets in IP vs FT enriched genes than in the IP vs IN comparison. A detailed list of the enriched genes in AGO2-IP vs FT and GW182-IP vs FT analyses is provided in Additional file 3, and an overview of their expression levels is shown in Additional file 4. We first noticed that the intersection between the two sets of enriched genes in AGO2 and GW182-IP showed a poor, yet significant, overlap, as shown in Additional file 5. Our list of enriched genes in the AGO2 IP vs FT comparison showed a statistically significant overlap with the published list of 616 enriched genes for AGO2-IP in MCF-7 cells [13]. Unfortunately, no high throughput analysis results are yet publicly available for any anti-GW182 antibody, which makes it impossible to perform a similar comparison for enriched genes in GW182-IP. The two sets of enriched/underrepresented genes, named UP/LOW_AGO2 and UP/LOW_GW182, were used, in the analysis described below, to select the features capable of distinguishing the mRNA associated with the AGO2 and GW182 proteins, respectively.

### Expression-based variables used for characterizing enriched genes in IP samples

**Fig. 2:**
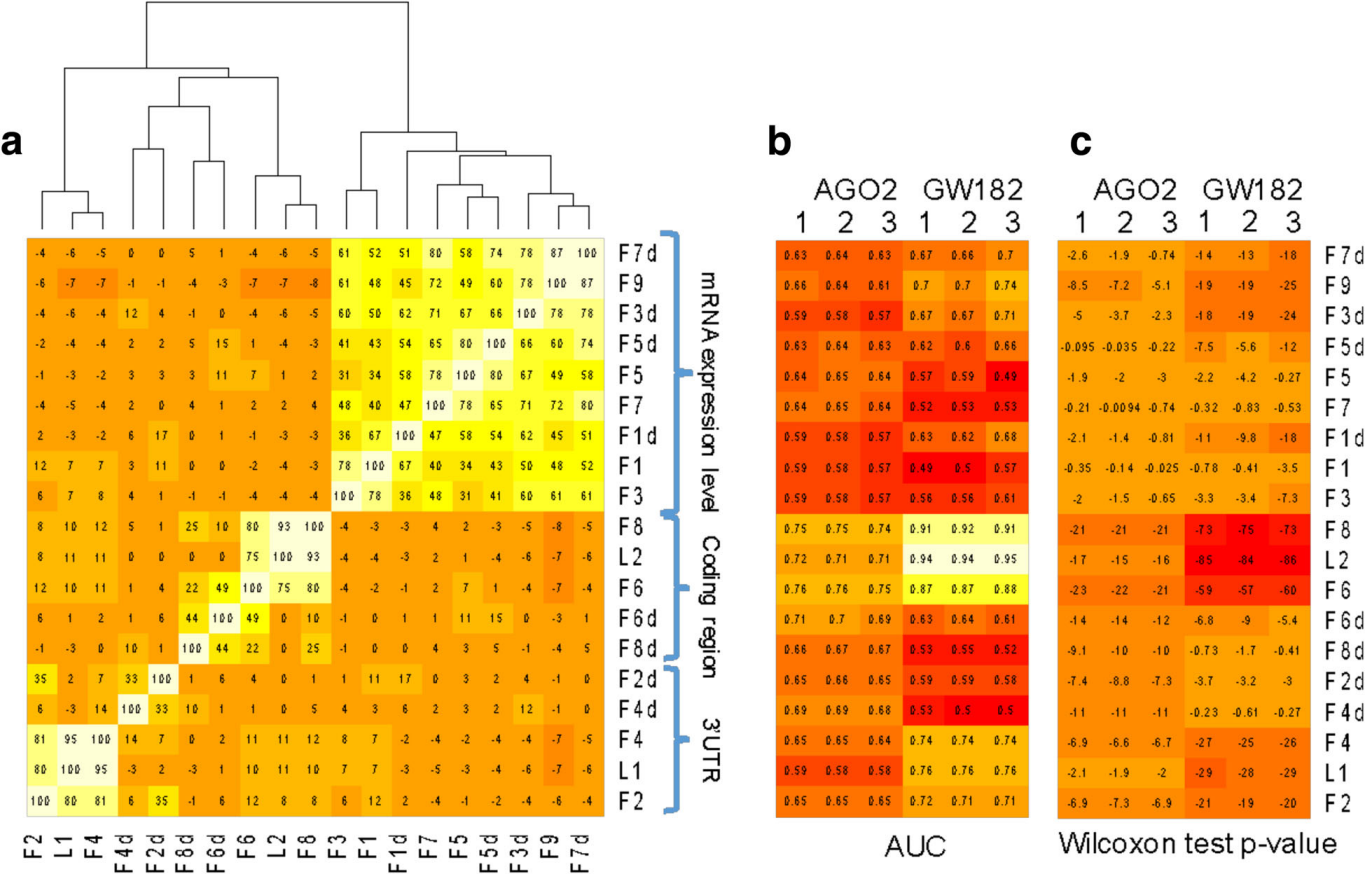
Behavior overview of variables listed in Table 1. **a** Heatmap representation of the correlation block matrix of the variables computed with AGO2_IN1 miRNA and mRNA expression profiles. The reported numbers are the correlation values, expressed in the range [− 100:100]. **b** ROC-AUC values obtained by classifying enriched/underrepresented genes associated with the variables computed with each IN expression profile. **c** Wilcoxon test *p*-values (log10) obtained by comparing the variable values associated with the enriched/underrepresented gene sets. In both **b**) and **c**), the variables computed with the three AGO2 IN profiles were used to distinguish enriched and underrepresented genes in AGO2-IP vs FT. The variables computed with the three GW182 IN profiles were used to distinguish enriched and underrepresented genes in GW182-IP vs FT.

To have better insight into the roles of the GW182 and AGO2 proteins in miRNA regulatory activity, and with the aim of selecting the most useful variables for distinguishing between enriched and underrepresented genes in IP samples, we tested formulas including mRNA and miRNA expression levels in IN samples and miRNA predicted binding sites on 3’UTR and coding regions of mRNAs. Specifically, we considered 19 variables, all computed by using features characterizing the mRNA sequences and IN sample gene expression. Table 1 describes all the considered variables. The defined variables display high correlations among each other, as shown in the correlation matrix reported in Fig. 2a, where variables are specifically computed for the AGO2_IN1 sample. Analogous results were obtained when using the expression profile information of other IN samples. Three main clusters of highly correlated variables were clearly visible, one that contains all the variables included in the formula for the mRNA expression profile, and the other two that relate to the presence of miRNA binding sites in the coding region and 3’UTR.

**Table 1:** Definition of variables used to model miRNA activity

Variable name	Formula	*BS*
F1	∑_*i*_*expr*(*miRNA*_*i*_) × *BS*_*ij*_ × *expr*(*mRNA*_*j*_)	number in 3’UTR
F2	∑_*i*_*expr*(*miRNA*_*i*_) × *BS*_*ij*_	number in 3’UTR
F3	∑_*i*_*BS*_*ij*_ × *expr*(*mRNA*_*j*_)	number in 3’UTR
F4	∑_*i*_*BS*_*ij*_	number in 3’UTR
F1d	∑_*i*_*expr*(*miRNA*_*i*_) × *dBS*_*ij*_ × *expr*(*mRNA*_*j*_)	density in 3’UTR
F2d	∑_*i*_*expr*(*miRNA*_*i*_) × *dBS*_*ij*_	density in 3’UTR
F3d	∑_*i*_*dBS*_*ij*_ × *expr*(*mRNA*_*j*_)	density in 3’UTR
F4d	∑_*i*_*dBS*_*ij*_	density in 3’UTR
F5	∑_*i*_*expr*(*miRNA*_*i*_) × *BS*_*ij*_ × *expr*(*mRNA*_*j*_)	number in coding region
F6	∑_*i*_*expr*(*miRNA*_*i*_) × *BS*_*ij*_	number in coding region
F7	∑_*i*_*BS*_*ij*_ × *expr*(*mRNA*_*j*_)	number in coding region
F8	∑_*i*_*BS*_*ij*_	number in coding region
F5d	∑_*i*_*expr*(*miRNA*_*i*_) × *dBS*_*ij*_ × *expr*(*mRNA*_*j*_)	density in coding region
F6d	∑_*i*_*expr*(*miRNA*_*i*_) × *dBS*_*ij*_	density in coding region
F7d	∑_*i*_*dBS*_*ij*_ × *expr*(*mRNA*_*j*_)	density in coding region
F8d	∑_*i*_*dBS*_*ij*_	density in coding region
F9	*expr*(*mRNA*_*j*_)	Not applicable
L1	length of 3’UTR	Not applicable
L2	length of coding region	Not applicable

The column ***BS*** provides details about the miRNA predicted binding sites used to compute *BS*_*ij*_ (the binding sites matrix). For each variable, the Formula defines the values associated to each *mRNA*_*j*_

### Enriched and underrepresented genes in anti-AGO2 RIP are efficiently distinguished by miRNA binding sites in mRNA coding regions weighted by miRNA expression

**Fig. 3:**
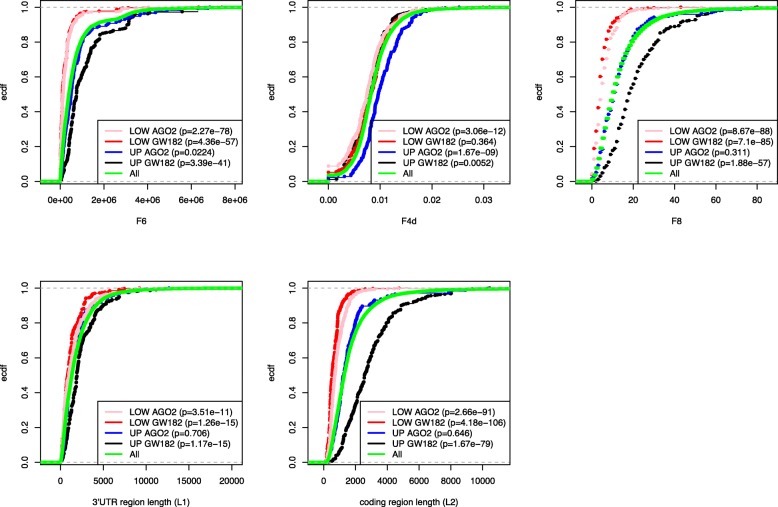
Graphic representation of selected features values associated to enriched and underrepresented genes. Empirical cumulative distribution function (ECDF) of F6, F4d, F8, L1 and L2 variables computed for enriched (UP) and underrepresented (LOW) genes in AGO2 IP vs FT and GW182 IP vs FT analyses. The reported p-values were obtained by performing a Wilcoxon-test comparing the values assumed by the selected set of genes with the values assumed by all the genes (16,363, green lines)

We first tested the performance of each of the 19 variables to distinguish the enriched genes (UP) in AGO2-IP vs FT from the underrepresented (LOW) genes. We computed the variables by using the expression profiles from each individual anti-AGO2 RIP experiment and performed a ROC analysis and a Wilcoxon test, using the UP/LOW genes detected comparing AGO2-IP vs FT as a reference set. Figure 2b and c show the obtained AUC values and the Wilcoxon-test *p*-values, both used as an estimation of performance in distinguishing UP genes from LOW genes. Similar results are shown in Additional file 6, where the binding sites were predicted by using different prediction tools. The Targetscan prediction tool showed the best performance in distinguishing the enriched genes. Thus, we decided to use it in any further analyses to compute BS matrices. It was evident that the features belonging to the cluster related to the coding region length were the most efficient. Indeed, F6 and F8 variables were the best variables for distinguishing between enriched and underrepresented genes in anti-AGO2 RIP samples. F8 counts the number of binding sites in the coding region of the mRNA, while the number of binding sites is weighted by the miRNA expression values in F6. Both F6 and F8 variables are highly correlated with the L2 variable, which could have been anticipated, since the longer the coding region is, the higher the number of binding sites detected in the region by any binding site prediction algorithm. Figure 3 clearly shows that F6, F8, and L2 variables assume lower values for LOW_AGO2 genes with respect to all genes. On the other hand, the variable with the next highest performance, not belonging to the L2 cluster, was the F4d variable. Figure 3 shows that F4d assumes higher values for UP_AGO2 with respect to all genes. The behavior of F4d promised to be synergistic with F6 in distinguishing UP and LOW genes, and, therefore, we further discuss it in a separate section.

**Fig. 4:**
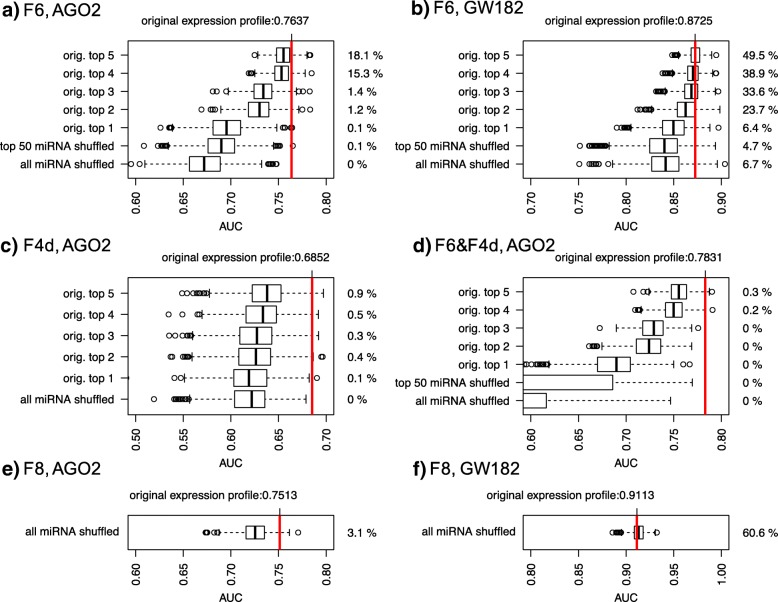
Graphic representation of the effect of miRNA expression profile shuffling. Each boxplot represents the AUC values obtained with 1000 simulated miRNA expression profiles. The percentage on the right of each boxplot refers to the number of times an AUC value was greater than the AUC obtained with the original miRNA expression profile (red vertical line). **a** Performance of simulated F6 variables in distinguishing AGO2 enriched/underrepresented genes. **b** Performance of simulated F6 variables in distinguishing GW182 enriched/underrepresented genes. **c** Performance of simulated F4d variables in distinguishing AGO2 enriched/underrepresented genes. **d** Performance of simulated SVM models (F6&F4d variables) in distinguishing AGO2 enriched/underrepresented genes. **e** Performance of simulated F8 variables in distinguishing AGO2 enriched/underrepresented genes. **f** Performance of simulated F8 variables in distinguishing GW182enriched/underrepresented genes

Next, we verified that the high performance of variables F6 and F8 was specifically due to the effects of the miRNA expression profile in the formula. Specifically, we considered 1000 simulated miRNA expression profiles, as obtained by assigning the original expression profile to 50 random miRNAs, chosen from among all the miRNAs expressed in the sample, and 1000 simulated miRNA expression profiles, as obtained by shuffling the original 50 miRNAs found to be highly expressed (top 50 expressed). The first block of simulations was less conservative, and its aim was to test whether the identity of the top 50 expressed miRNAs was determinant for reaching the original performance; it was the only block of simulations meaningful for testing the performance of the F8 variable. The second block of simulations was more conservative, and its aim was to assess whether the specific expression profile associated with the top 50 miRNAs was determinant. In both cases, the performance of the simulated F6 and F8 variables was significantly lower than the F6 and F8 variables obtained by including the original miRNA expression profile (see Fig. 4a). We also tested simulations that were more conservative by holding the expression profile of the highly expressed miRNAs fixed while shuffling the expression of the remaining ones. Figure 4a shows the results of these simulations obtained by fixing up to five top expressed miRNAs, and Additional file 7 contains the results obtained by serially holding all the top 50 miRNAs fixed. As the number of the top expressed miRNAs increased, the F6 variable performance became closer to that obtained with the original miRNA expression profile; in addition, the higher the number of miRNAs fixed, the closer it got to the original performance level. As a result, we concluded that the miRNA expression profile is crucial for distinguishing AGO2-associated miRNA targets, especially the expression profile of the first top expressed miRNAs, and that the most relevant miRNA binding sites are the ones found in the coding region.

### Enriched and underrepresented genes in anti-GW182 RIP are efficiently distinguished by coding region length

The performance of each of the 19 variables was tested to distinguish between the enriched genes in GW182-IP vs FT and the underrepresented ones. Figure 2b and c show that the features belonging to the cluster related to the coding region length are the most efficient at distinguishing between enriched genes in anti-GW182 RIP samples. In this case, the best feature for distinguishing the enriched genes in GW182-IP samples was the coding region length of the mRNA, i.e., the L2 variable, with a surprisingly very high performance (average AUC > 0.9). The average AUC associated with the F6 variable was also very high (average AUC = 0.87); however, the miRNA expression profile was not crucial for reaching such high performance since a shuffled expression profile was not significantly deficient in distinguishing the enriched genes (Fig. 4b, Additional file 7). In Fig. 3, we compare the ECDF of the coding region length of the UP and LOW genes in the anti-GW182 RIP experiments. The separation between UP and LOW genes in anti-GW182 RIP samples is evident in the coding region length values, though less in the 3’UTR length values. Wilcoxon tests were performed to compare the 3’UTR and coding region length of GW182_UP and DOWN genes with all gene lengths, and gave highly significant *p*-values. Anti-GW182 RIP gene expression profiles, which could be used to support our hypothesis that the mRNA coding region length is a relevant feature for GW182 activity, are not available, and none of the enriched group of genes reported in the literature regards breast cancer cells. Nevertheless, we considered the IP-enrichment results of 7820 genes published by Landthaler and collaborators [14], where the authors generated HEK293 cell lines stably expressing epitope-tagged human AGO and GW proteins and used such cells to detect enriched mRNA in miRNA-containing ribonucleoprotein particles through a microarray analysis. They found a high overlap among the enriched targets of the AGO and GW182 family proteins by analyzing the top immunoprecipitated transcripts associated with the four AGO proteins vs the ones associated with the three GW182 proteins. Differently from [14], we considered the non-overlapping enriched genes, and we found that the mRNAs enriched only in GW182-IP had significantly longer 3’UTR and coding regions (see Additional file 8).

### SVM models improve performance in distinguishing enriched genes

**Fig. 5:**
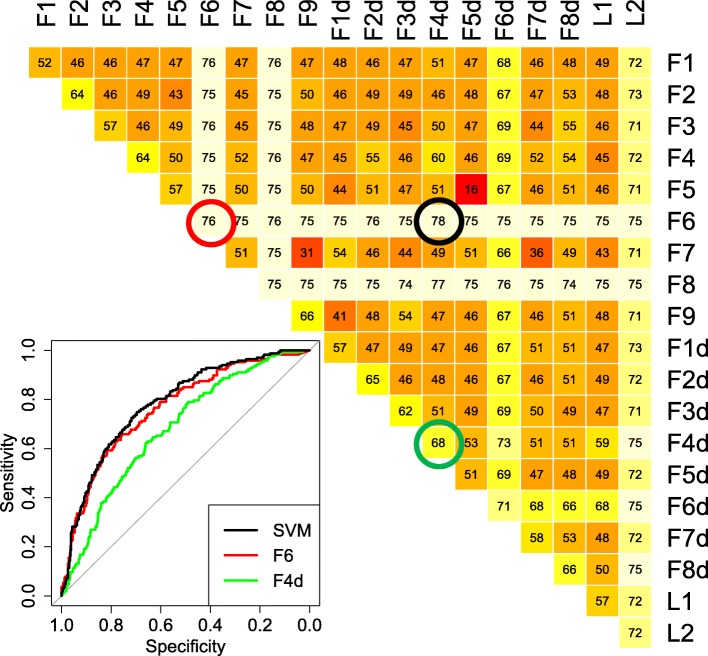
Support Vector Machine models performance summary. AUC values of SVM models trained with any pair of variables defined in Table 1, used to classify enriched/underrepresented genes in AGO2-IP vs FT comparison. Variables were computed by using the AGO2_IN1 expression profiles. Values are in the range [0:100]. Values in the diagonal refer to single variable performance. The ROC plot at bottom left represents the results obtained with the best-performing SVM model (F6&F4d, black line) and with the two single variables, F6 (red line) and F4d (green line)

We tested whether a combination of two variables could significantly improve the classification of the performance of enriched/underrepresented genes. An SVM algorithm model was trained with each pair of features, and the AUC results for each pair are reported in Fig. 5. The best performance in predicting AGO2-bound mRNAs was associated with the F6-F4d variable pair, with an AUC significantly higher than the one obtained with F6 only (AUC = 0.78; DeLong’s test *p*-value < 0.05). The F4d variable takes into account the density of the binding sites in the 3’UTR, as predicted for the top 50 expressed miRNAs. The F4d variable performance by itself (AUC = 0.68) is the highest among the features not highly correlated with the F6 variable. We checked whether the identity of the top 50 expressed miRNAs was crucial for reaching such a performance by randomly changing the identity of the 50 miRNAs in the F4d formula, and holding the expression of an increasing number of top miRNAs fixed. The results are plotted in Fig. 4c and Additional file 7, and they show that, when using randomly chosen miRNAs, the performance is significantly lower than the one obtained with the true top 50 expressed miRNAs. Differently from what was obtained for the F6 variable, to reach the performance obtained with the original miRNA expression profile, the expression of almost all the miRNAs had to be held, meaning that the identity of the top 50 miRNAs is substantially important to the F4d variable’s performance.

Analogous simulations were done for the predictions obtained with the SVM model trained with the F6 and F4d variables, shown in Fig. 4d and Additional file 7. The results show that several miRNAs had to be fixed in order to reach a performance similar to that obtained with the original miRNA expression profile. Finally, we tested how slightly different expression profiles, such as the ones obtained by experiment replica, may affect enriched/underrepresented gene classification. Specifically, we used an SVM model trained with features computed with miRNA expression profiles from one IN sample to classify genes with higher vs lower IP/FT ratio, computed in each of the three experiments (see Additional file 9). Our results show that higher performance was always obtained when predictions of IP/FT ratio values in one experiment were obtained with the miRNA expression profile belonging to the IN sample expression profile of the same experiment.

The pair of variables that best predicted the GW182-bound mRNAs was the L1 and L2 pair, i.e., the length of the 3’UTR and the coding region, respectively, but the improvement in the AUC value was not statistically significant (DeLong’s test *p*-value > 0.05).

## Discussion

We analyzed the activity of two endogenous interacting proteins, AGO2 and GW182, in MCF-7 cell cytoplasm. Both are involved in RISCs, and we analyzed the RNA co-immunoprecipitated with the selected proteins, which was expected to be enriched in genes involved in endogenous miRNA regulatory activity. Data from RIP-Chip experiments served to model miRNA activity by assigning variables based on miRNA expression profiles to each mRNA target, searching for the ones that would better distinguish the enriched genes in RIP samples. We expected that the detected variables could reveal which information was relevant for modeling miRNA activity and the RISC proteins’ roles.

Our results show that mRNAs co-immunoprecipitated with the two proteins have different characteristics. Such a finding might appear in contrast with a previous analysis performed in HEK293 cell lines, in which tagged-AGO2 or tagged-GW182/TNRC6A proteins were stably overexpressed and the AGO protein family and the GW182 protein family were found to be associated with highly similar sets of transcripts [14]. The low consistency with this previous study might indicate a different composition of RISCs in MCF-7 cells than HEK293 cells. Moreover, analysis under physiological conditions vs overexpressed AGO or GW182 might also explain the differences, and the fact that the authors analyzed the top immunoprecipitated transcripts for the whole AGO family (AGO1–4) vs the GW182 family (TNRC6A-C) might have mitigated RNA enrichment differences with respect to what we obtained through the comparison of two specific proteins, i.e., AGO2 and TNRC6A. Indeed, it has been reported that AGO1 and AGO2 proteins interact with a distinct set of miRNAs [24] and, as a consequence, with different mRNA targets, whereas the GW182/TNRC6A protein interacts with the whole AGO protein family [2]. This evidence also justifies the high similarity we found between the miRNA expression profiles of GW182-IP and FT, in contrast with more specific miRNA expression profiles associated with the AGO2-IP and FT samples (Fig. 1d). Furthermore, although a high degree of redundancy among the members of each protein family has been reported, it cannot be excluded that the use of different GW182 antibodies and/or slightly different experimental conditions, e.g., buffer stringency, might result in a different enrichment of RNAs in the immunoprecipitated samples. To this end, a systematic analysis of the data obtained using the same antibody in the same cell background, or the use of methods based on biochemical approaches, like the one described by Hauptmann and coworkers [16], might definitively clear up this point.

We found that the mRNAs co-immunoprecipitated with the AGO2 protein can be distinguished from the underrepresented mRNAs by considering the number of miRNA binding sites in the coding region, weighted by miRNA expression level. In order to improve the classification performance, we also trained an SVM with two features at a time, and we found that the additional feature to be considered was the density of the binding sites predicted in the 3’UTR of mRNA. We then performed simulations by shuffling the miRNA expression profiles in order to detect which miRNAs are relevant to composing the features used to distinguish enriched and underrepresented genes. When the performance obtained by randomly shuffling a set of miRNAs is significantly lower than the performance obtained with the original miRNA expression profile, we can assess that the set of miRNAs replaced is relevant in the classification. Results show that the only relevant miRNAs, when considering binding sites in the mRNA coding regions, are the top two to three of those expressed. On the contrary, almost all of the top 50 expressed miRNAs are relevant when considering the binding sites in the 3’UTR of mRNA, with a prominent exception being the top expressed one, i.e., hsa-miR-21-5p. The expression level detected for hsa-miR-21-5p is very high, by itself covering 60% of the total miRNA expression profile, and we suppose that its distinctive behavior is related to saturation effects in miRNA activity, which we plan to investigate in further studies.

In addition to simulated miRNA expression profiles, we tested how switching miRNA expression profiles across our experimental replicates affects the performance of the classification algorithm. We found that even slight differences in the expression profiles of the single replicate IN samples gave rise to differences in enriched vs underrepresented gene classification, leading to the conclusion that the combination of mRNA and miRNA expression profiles from the same experiment gives the best performance.

On the other hand, we clearly observed that the mRNA co-immunoprecipitated with the GW182 protein was highly enriched with genes with longer coding regions. In this case, enriched/underrepresented gene classification does not depend on the miRNA expression profile, but only on 3’UTR and coding region lengths. We confirmed this result by analyzing the data from Landthaler and coworkers [14]. Our interpretation is that GW182 complexes preferentially sequester the longer mRNAs in the process of populating GW/P-bodies.

While functionally diverse RISCs lacking GW182 have been described [32], the interaction between mRNAs and GW182 is reported to be mediated by the miRNA and AGO proteins and, so far, no direct interaction has been demonstrated between GW182 and mRNA. Recently, Elkayam and coauthors [33] showed that, differently from AGO proteins, which have a single GW182-binding site, GW182 can recruit up to three copies of AGO proteins via its three distinct GW motifs. We believe that such a feature supports our results, since the longer the mRNA is, the higher the number of miRNA binding sites and the probability that RNA-loaded AGO proteins would find cooperative binding sites within the right distance to interact with the same GW182 protein. In this case, the model of single binding sites weighted by miRNA expression profile is probably oversimplified, and further analysis is required to include collaboration effects. To our knowledge, the involvement of mRNA length in GW182 recruitment is a novel observation that may contribute to shedding light on the different activities of the AGO2 and GW182 proteins in various RISCs and/or in diverse cellular districts such as GW/P-bodies.

## Conclusions

In this work, we aimed to unravel RISC activity by analyzing a novel RIP-Chip data set obtained by the immunoprecipitation of two RISC proteins, AGO2 and GW182. We analyzed the overexpressed genes in the anti-AGO2 and anti-GW182 RIP samples vs the respective FT samples, and we revealed different features characterizing the enriched genes in the two data sets.

AGO2-associated mRNAs are characterized by a high number of binding sites in the coding region for top expressed miRNAs and by a high density of binding sites in the 3’UTR region. On the other hand, GW182-associated mRNAs are characterized by long coding regions. These different characteristics may underline the different roles played by the selected proteins in the RISC machinery activity. Our data confirm that the anti-AGO2 RIP gives an accurate picture of which RNA is involved in miRNA regulatory activity. Regarding the anti-GW182 RIP, data show no significant involvement of miRNA expression profiles in GW182-associated mRNA selection, at least within a simplified model of single binding sites weighted by miRNA expression profile. Our results support the hypothesis that, after being recruited by the miRNA machinery, only the mRNAs with longer coding regions are destined to be stored in GW/P bodies, while shorter mRNAs are most likely processed in different ways that lead to degradation rather than storage.

## Additional files

Additional file 1: Analysis of miRNA expression in AGO2 and GW182-IP samples. a) miRNA expression level in AGO2-IP samples (average value from the three performed experiments) vs the expression level in IN samples (average value from the three performed experiments). The Pearson correlation values reported on the top of the picture were computed by using all the expressed miRNA, and the top 100 or 50 expressed miRNAs. The colored points refer to miRNA that have been validated by RT-PCR data. Green points refer to hsa-miR-141-3p, hsa-miR-21-5p, hsa-let-7f-5p, hsa-miR-16-5p, hsa-miR-24-3p, hsa-miR-27a-3p, hsa-miR-23a-3p. The red point refers to hsa-miR-1260a. b) Comparison of IP/IN ratios obtained by RT-PCR data (normalized by IgG control data) and microarray data (normalized by Quantile normalization). The underrepresentation of hsa-miR-1260a was confirmed by RT-PCR. c) and d) Analysis of GW182-IP samples performed as described in a) and b). (PDF 47 kb)

Additional file 2: Gene set enrichment analysis results with seven top expressed miRNA predicted targets sets. Predicted targets of miRNAs (column 1) were predicted with three different target prediction tools (column 2). The total number of predicted targets is indicated in column 3. Five lists of genes were analyzed. For each list of genes the number of genes in common with the predicted targets and the associated hypergeometric test pvalue are provided. The total number of genes considered in the analysis is 16,392. The five considered lists are: a list of genes enriched in AGO2 IP sample from [13]; lists of genes enriched in AGO2 IP vs IN and IP vs FT samples; lists of genes enriched in GW182 IP vs IN and IP vs FT samples. (XLS 64 kb)

Additional file 3: Summary of enriched and underrepresented genes. Summary of enriched and underrepresented genes in AGO2 and GW182-IP vs FT comparisons performed by SAMR (column 2–3). The enrichment results obtained with the REA algorithm are reported in columns 4–5. Columns 6 and 7 report the 3’UTR and Coding region (CR) lengths respectively. In columns 8–21 we report the number of binding sites predicted by Targetscan in the 3’UTR and the Coding region of seven highly expressed miRNAs. (XLS 294 kb)

Additional file 4: Overview of gene expression levels in IP and FT samples. Focus on the enriched genes in AGO2-IP and GW182-IP vs FT samples. The reported expression levels are computed as the average values of the three performed experimental replicates. (PDF 1423 kb)

Additional file 5: Venn diagram of lists of enriched genes. The considered lists are: AGO2-IP (UP_AGO2 set), the list of enriched genes detected by Fan et al. [13] (UP_AGO2_Fan) and our list of enriched genes in GW182-IP sample (UP_GW182). The reported *p*-values refer to the closest intersection set of genes and are computed with one tail Fisher-test. (PDF 34 kb)

Additional file 6: Wilcoxon test p-values summary. Wilcoxon test p-values (log10) obtained by comparing the variable values associated with the enriched/underrepresented genes sets. Three different miRNA target prediction tools (Targetscan, PITA and miRanda) were used to compute the necessary binding sites (BS) matrices. The BS matrices used to compute the p-values in the last panel were obtained by considering BS predicted by at least two of the three prediction tools. In each panel, the variables computed with the three AGO2 IN profiles were used to distinguish enriched and underrepresented genes in AGO2-IP vs FT and the variables computed with the three GW182 IN profiles were used to distinguish enriched and underrepresented genes in GW182-IP vs FT. (PDF 133 kb)

Additional file 7: Summary of miRNA expression profiles shuffling effects. ROC analysis was performed to evaluate the performance of F6 and F4d variables, computed with simulated miRNA profiles, in distinguishing enriched/underrepresented genes in AGO2 or GW182-IP samples. Each panel reports the AUC values obtained with simulated variables. Each boxplot refers to AUC values obtained with a specific set of simulations, where the expression profile of a set of miRNAs was shuffled. The boxplot in the center was obtained by shuffling all miRNAs. The boxplots from the center to the right refer to simulations where all the miRNAs were shuffled with the exception of n top expressed miRNAs, n increasing in the right direction. The boxplots from the center to the left refer to simulations where all the miRNAs were shuffled with the exception of n low expressed miRNAs, n increasing in the left direction. The green horizontal line defines the AUC value obtained with the original miRNA expression profile. Boxplots are colored in red if less than 5% of the simulations reach the AUC original value, green otherwise. This file contains the following simulation results: A. Simulated F6 variable distinguishing AGO2 enriched vs underrepresented genes. B. Simulated F6 variable distinguishing GW182 enriched vs underrepresented genes. C. Simulated F4d variable distinguishing AGO2 enriched vs underrepresented genes. D. Simulated F6&F4d SVM model distinguishing AGO2 enriched vs underrepresented genes. (PDF 555 kb)

Additional file 8: Empirical Cumulative Distribution Function of 3’UTR and coding region length of IP-Enriched genes. Enriched genes in AGO (1–4) and in GW182 protein family IP selected by considering log2 IP-Enrichment of transcript greater than 1. Data are downloaded from Landthaler et al. [14]. The Empirical Cumulative Distribution Function of the 3’UTR length (top) and coding region length (bottom) of genes enriched exclusively by AGO-IP (red line), GW182-IP (blue line) and both IPs (black line) are reported. The reported *p*-value is computed by performing a Wilcoxon test to compare the length distributions of genes enriched exclusively in AGO-IP and in GW182-IP. (PDF 145 kb)

Additional file 9: Summary of miRNA expression profiles switch between experiment replicas. ROC analysis of F6&F4d SVM model trained with variables calculated with miRNA expression profiles from each of the three anti-AGO2 RIP experiments. SVM models were used to classify the top 1000 and the bottom 1000 genes with respect to the IP/FT mRNA expression ratio, computed for each of the three AGO2 RIP experiments. (PDF 653 kb)

## Abbreviations

3’UTR: 3′ untranslated regions
AUC: Area under the (ROC) curve
ECDF: Empirical Cumulative Distribution Function
FT: Flow-through
miRNA: microRNA
RIP – Chip: RIP coupled to microarray
RIP: RNA-binding protein immunoprecipitation
RISC: RNA-induced silencing complex
ROC: Receiver operating characteristic
SVM: Support vector machine
