# Supplementary figures and images for: RIP-Chip analysis supports different roles for AGO2 and GW182 proteins in recruiting and processing microRNA targets
Source: BMC Bioinformatics. 2019 Apr 18;20(Suppl 4):120. doi: 10.1186/s12859-019-2683-y (PMC6471694; doi:10.1186/s12859-019-2683-y)

**corr(all) = 0.667 ; corr(top100) = 0.681 ; corr(top50) = 0.605**

**a**

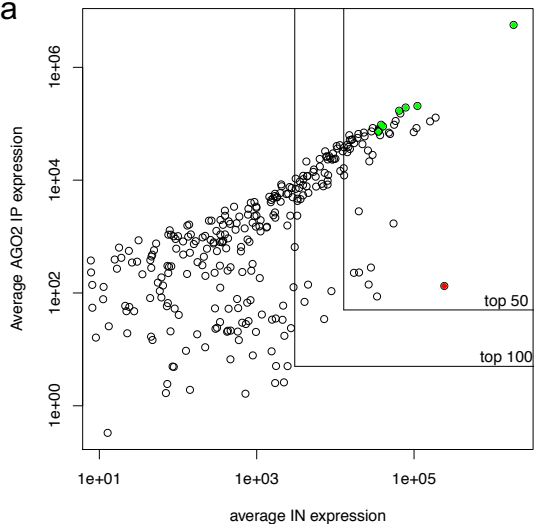

**b**

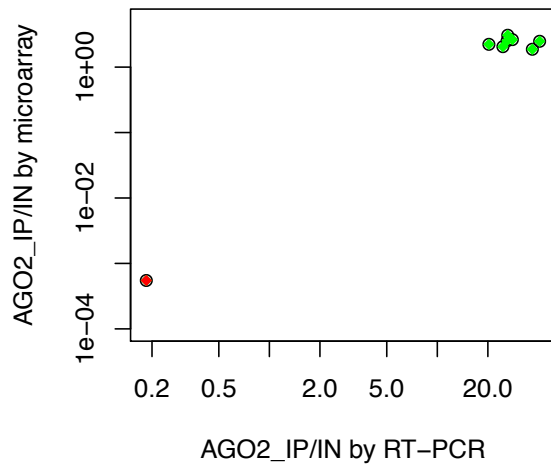

**corr(all) = 0.777 ; corr(top100) = 0.77 ; corr(top50) = 0.702**

**c**

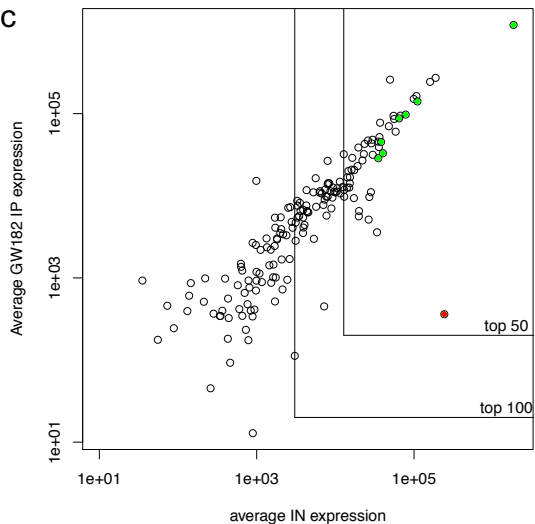

**d**

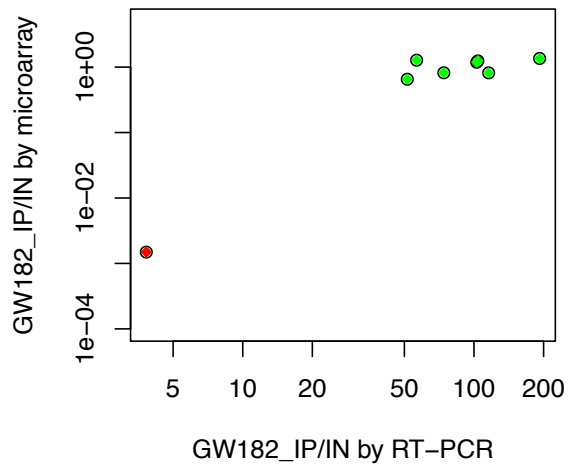

Supplement: Supplementary file 1 — Analysis of miRNA expression in AGO2 and GW182-IP samples. a) miRNA expression level in AGO2-IP samples (average value from the three performed experiments) vs the expression level in IN samples (average value from the three performed experiments). The Pearson correlation values reported on the top of the picture were computed by using all the expressed miRNA, and the top 100 or 50 expressed miRNAs. The colored points refer to miRNA that have been validated by RT-PCR data. Green points refer to hsa-miR-141-3p, hsa-miR-21-5p, hsa-let-7f-5p, hsa-miR-16-5p, hsa-miR-24-3p, hsa-miR-27a-3p, hsa-miR-23a-3p. The red point refers to hsa-miR-1260a. b) Comparison of IP/IN ratios obtained by RT-PCR data (normalized by IgG control data) and microarray data (normalized by Quantile normalization). The underrepresentation of hsa-miR-1260a was confirmed by RT-PCR. c) and d) Analysis of GW182-IP samples performed as described in a) and b). (PDF 47 kb) [file 12859_2019_2683_MOESM1_ESM.pdf]

**AGO2 RIP**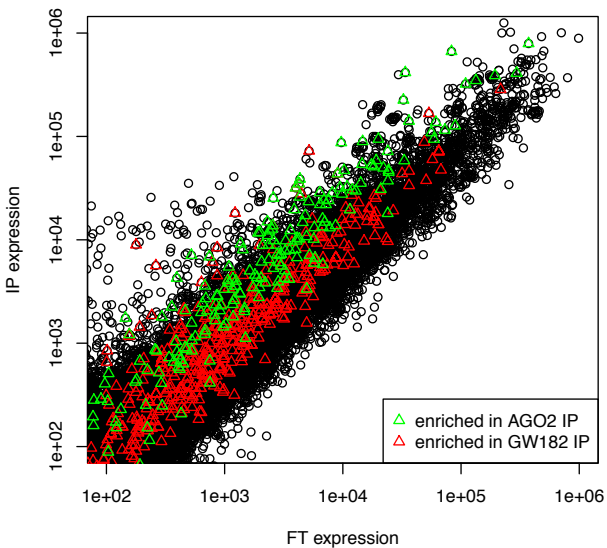**Expression in IPs**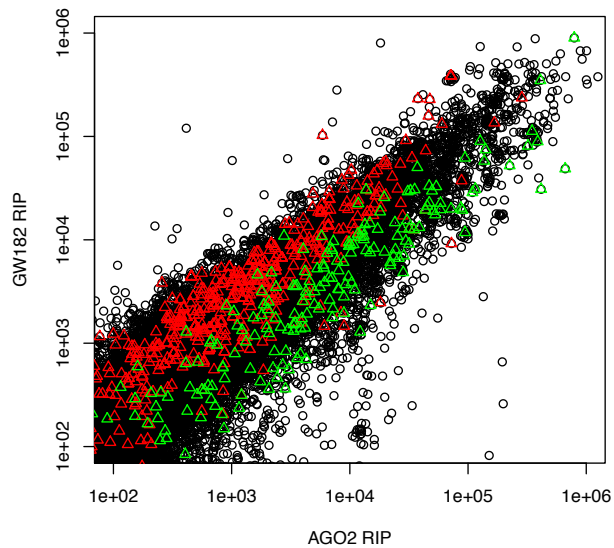**GW182 RIP**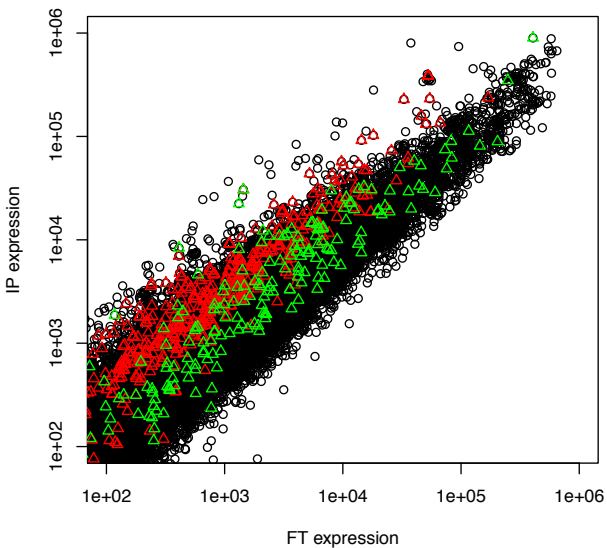**Expression in FTs**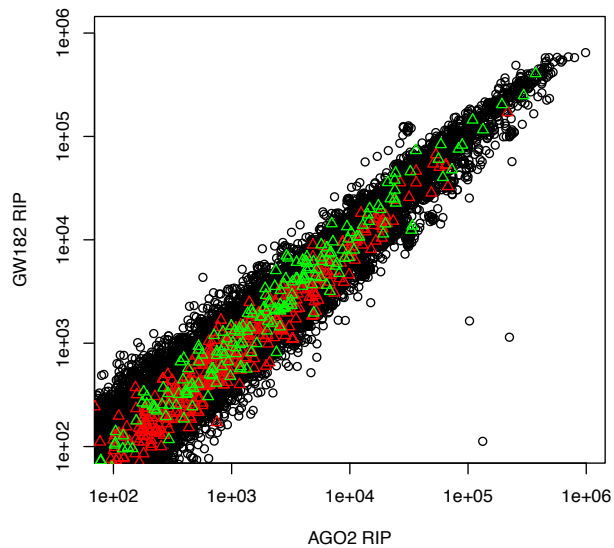

Supplement: Supplementary file 4 — Overview of gene expression levels in IP and FT samples. Focus on the enriched genes in AGO2-IP and GW182-IP vs FT samples. The reported expression levels are computed as the average values of the three performed experimental replicates. (PDF 1423 kb) [file 12859_2019_2683_MOESM4_ESM.pdf]

Universe = 16363 genes

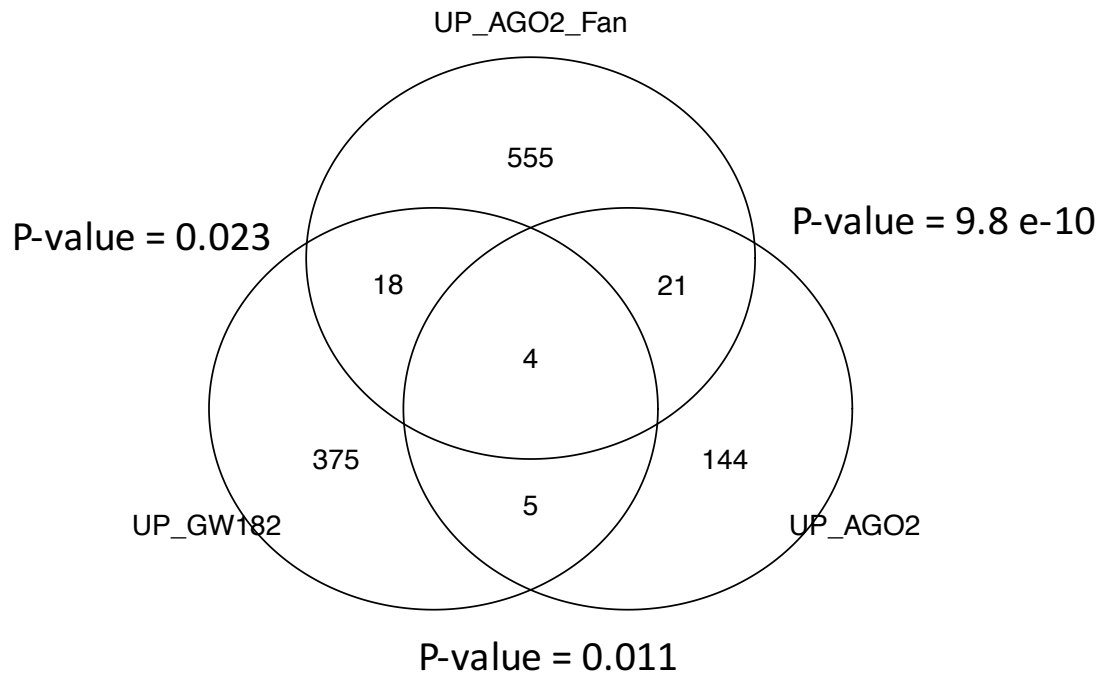

Supplement: Supplementary file 5 — Venn diagram of lists of enriched genes. The considered lists are: AGO2-IP (UP_AGO2 set), the list of enriched genes detected by Fan et al. [13] (UP_AGO2_Fan) and our list of enriched genes in GW182-IP sample (UP_GW182). The reported p-values refer to the closest intersection set of genes and are computed with one tail Fisher-test. (PDF 34 kb) [file 12859_2019_2683_MOESM5_ESM.pdf]

# IP-Enriched genes behavior

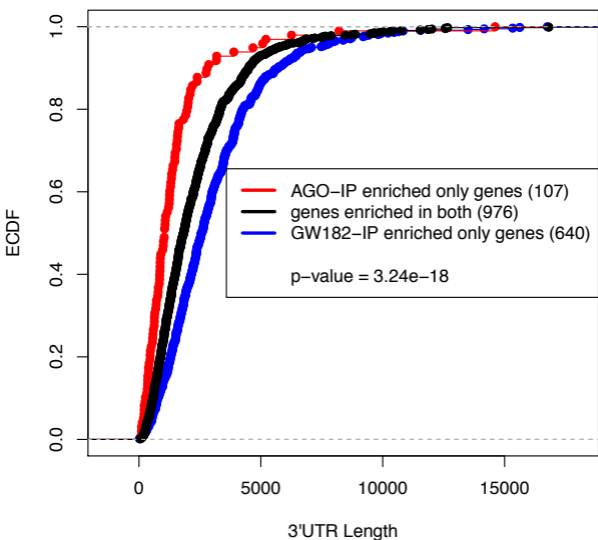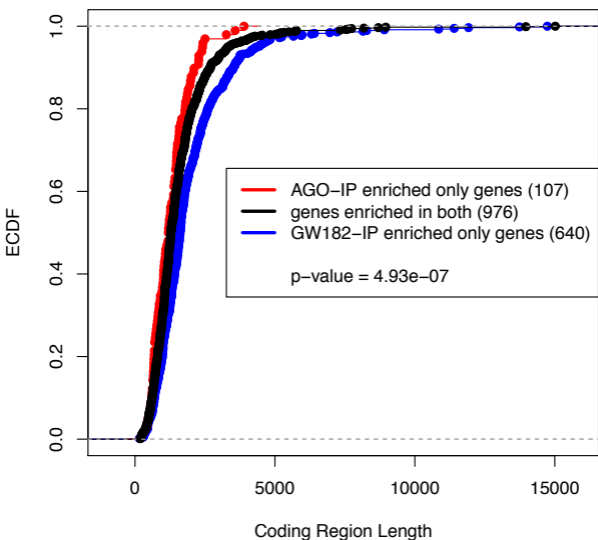

Supplement: Supplementary file 8 — Empirical Cumulative Distribution Function of 3’UTR and coding region length of IP-Enriched genes. Enriched genes in AGO (1–4) and in GW182 protein family IP selected by considering log2 IP-Enrichment of transcript greater than 1. Data are downloaded from Landthaler et al. [14]. The Empirical Cumulative Distribution Function of the 3’UTR length (top) and coding region length (bottom) of genes enriched exclusively by AGO-IP (red line), GW182-IP (blue line) and both IPs (black line) are reported. The reported p-value is computed by performing a Wilcoxon test to compare the length distributions of genes enriched exclusively in AGO-IP and in GW182-IP. (PDF 145 kb) [file 12859_2019_2683_MOESM8_ESM.pdf]
