# Supplementary material for: RIP-Chip analysis supports different roles for AGO2 and GW182 proteins in recruiting and processing microRNA targets
Source: BMC Bioinformatics. 2019 Apr 18;20(Suppl 4):120. doi: 10.1186/s12859-019-2683-y (PMC6471694; doi:10.1186/s12859-019-2683-y)
Supplement: Supplementary file 6 — Wilcoxon test p-values summary. Wilcoxon test p-values (log10) obtained by comparing the variable values associated with the enriched/underrepresented genes sets. Three different miRNA target prediction tools (Targetscan, PITA and miRanda) were used to compute the necessary binding sites (BS) matrices. The BS matrices used to compute the p-values in the last panel were obtained by considering BS predicted by at least two of the three prediction tools. In each panel, the variables computed with the three AGO2 IN profiles were used to distinguish enriched and underrepresented genes in AGO2-IP vs FT and the variables computed with the three GW182 IN profiles were used to distinguish enriched and underrepresented genes in GW182-IP vs FT. (PDF 133 kb) [file 12859_2019_2683_MOESM6_ESM.pdf]

Targetscan

	AGO2 1	AGO2 2	AGO2 3	GW182 1	GW182 2	GW182 3
F7d	−2.6	−1.9	−0.79	−13	−13	−18
F9	−8.5	−7.2	−5.1	−19	−19	−25
F3d	−4.9	−3.7	−2.3	−18	−19	−24
F5d	−0.089	−0.024	−0.23	−7.4	−5.6	−12
F5	−1.9	−2	−3	−2.2	−4.2	−0.29
F7	−0.23	−0.0015	−0.71	−0.36	−0.85	−0.52
F1d	−2	−1.3	−0.79	−11	−9.8	−18
F1	−0.34	−0.13	−0.031	−0.74	−0.4	−3.4
F3	−2	−1.5	−0.64	−3.3	−3.3	−7.3
F8	−21	−21	−21	−74	−75	−73
L2	−17	−15	−16	−85	−84	−86
F6	−23	−22	−21	−59	−57	−60
F6d	−14	−14	−12	−6.8	−9	−5.4
F8d	−8.7	−10	−9.6	−0.98	−1.8	−0.51
F2d	−7.4	−8.9	−7.3	−3.7	−3.3	−3
F4d	−11	−11	−11	−0.25	−0.55	−0.21
F4	−7	−6.7	−6.7	−27	−25	−26
L1	−2.1	−1.9	−2	−29	−28	−29
F2	−6.9	−7.3	−6.9	−21	−19	−20

PITA

	AGO2 1	AGO2 2	AGO2 3	GW182 1	GW182 2	GW182 3
F7d	−2	−1.2	−0.37	−10	−10	−15
F9	−7.9	−6.7	−4.7	−19	−19	−25
F3d	−4.4	−3.5	−1.8	−23	−23	−30
F5d	−0.92	−0.61	−0.24	−6.2	−6.3	−9.8
F5	−0.43	−0.64	−1.3	−1.8	−2.8	−0.31
F7	−0.068	−0.16	−1.1	−0.4	−0.92	−0.58
F1d	−3	−2.4	−1.6	−17	−16	−25
F1	−0.99	−0.76	−0.38	−3.1	−2.1	−7.3
F3	−2	−1.5	−0.54	−6.2	−5.6	−11
F8	−20	−19	−22	−53	−52	−54
L2	−15	−14	−15	−83	−84	−85
F6	−16	−15	−19	−49	−50	−49
F6d	−9.3	−8.7	−11	−4.9	−3.7	−5.6
F8d	−12	−12	−14	−0.047	−0.15	−0.029
F2d	−3.9	−3.5	−3.8	−0.3	−0.064	−0.79
F4d	−3.7	−3.4	−4.1	−9	−8.3	−8.1
F4	−5.4	−4.8	−5.9	−10	−10	−10
L1	−2	−1.9	−1.9	−30	−30	−29
F2	−4	−3.8	−4.3	−13	−13	−12

miRanda

	AGO2 1	AGO2 2	AGO2 3	GW182 1	GW182 2	GW182 3
F7d	−2.9	−1.9	−0.96	−11	−13	−18
F9	−8.3	−6.7	−4.9	−17	−18	−24
F3d	−4.6	−3.4	−2.2	−18	−19	−25
F5d	−1.2	−0.95	−0.47	−7.1	−6.5	−11
F5	−0.48	−0.59	−1.2	−1.8	−2.8	−0.26
F7	−0.28	−0.038	−0.64	−0.52	−0.8	−0.76
F1d	−3.4	−2.7	−1.7	−12	−9.5	−18
F1	−0.97	−0.73	−0.3	−0.8	−0.26	−3.2
F3	−1.8	−1.3	−0.57	−3.4	−3.2	−7.2
F8	−22	−20	−22	−70	−70	−66
L2	−17	−15	−16	−83	−83	−85
F6	−15	−14	−14	−47	−47	−46
F6d	−5.9	−5.6	−5.7	−4.4	−4.6	−3.8
F8d	−7.9	−8.4	−8.8	−0.22	−0.32	−0.61
F2d	−2.5	−2.9	−3	−2	−2.7	−0.58
F4d	−9.1	−8.4	−8.1	−0.88	−1.7	−1.8
F4	−6.7	−6.1	−6.3	−22	−22	−22
L1	−2.4	−2	−2.1	−27	−27	−28
F2	−3.3	−3.4	−3.5	−19	−18	−17

intersection

	AGO2 1	AGO2 2	AGO2 3	GW182 1	GW182 2	GW182 3
F7d	−2.2	−1.8	−0.61	−11	−13	−16
F9	−7.9	−6.5	−4.7	−18	−18	−24
F3d	−4.4	−3.1	−2.2	−20	−21	−27
F5d	−0.087	−0.068	−0.2	−6.1	−6	−9.8
F5	−1.8	−1.9	−2.9	−1.8	−2.9	−0.34
F7	−0.095	−0.049	−0.91	−0.28	−0.37	−0.84
F1d	−2	−1.4	−1	−12	−11	−20
F1	−0.47	−0.23	−0.093	−1.2	−0.74	−4.3
F3	−2.1	−1.4	−0.75	−4.7	−4.5	−9.5
F8	−22	−21	−22	−64	−67	−61
L2	−16	−15	−16	−82	−82	−84
F6	−21	−20	−20	−44	−44	−47
F6d	−11	−10	−9.4	−2.2	−3.2	−1.5
F8d	−5.5	−6.6	−6.4	−3.8	−3.4	−5
F2d	−5.8	−5.8	−5.1	−0.83	−0.97	−0.03
F4d	−8.4	−8.3	−6.3	−5.5	−7.5	−7.9
F4	−7.6	−7.1	−6.5	−20	−20	−19
L1	−1.9	−1.8	−1.9	−28	−28	−28
F2	−5.6	−5.6	−5.1	−17	−16	−15
