# Supplementary material for: RIP-Chip analysis supports different roles for AGO2 and GW182 proteins in recruiting and processing microRNA targets
Source: BMC Bioinformatics. 2019 Apr 18;20(Suppl 4):120. doi: 10.1186/s12859-019-2683-y (PMC6471694; doi:10.1186/s12859-019-2683-y)
Supplement: Supplementary file 7 — Summary of miRNA expression profiles shuffling effects. ROC analysis was performed to evaluate the performance of F6 and F4d variables, computed with simulated miRNA profiles, in distinguishing enriched/underrepresented genes in AGO2 or GW182-IP samples. Each panel reports the AUC values obtained with simulated variables. Each boxplot refers to AUC values obtained with a specific set of simulations, where the expression profile of a set of miRNAs was shuffled. The boxplot in the center was obtained by shuffling all miRNAs. The boxplots from the center to the right refer to simulations where all the miRNAs were shuffled with the exception of n top expressed miRNAs, n increasing in the right direction. The boxplots from the center to the left refer to simulations where all the miRNAs were shuffled with the exception of n low expressed miRNAs, n increasing in the left direction. The green horizontal line defines the AUC value obtained with the original miRNA expression profile. Boxplots are colored in red if less than 5% of the simulations reach the AUC original value, green otherwise. This file contains the following simulation results: A. Simulated F6 variable distinguishing AGO2 enriched vs underrepresented genes. B. Simulated F6 variable distinguishing GW182 enriched vs underrepresented genes. C. Simulated F4d variable distinguishing AGO2 enriched vs underrepresented genes. D. Simulated F6&F4d SVM model distinguishing AGO2 enriched vs underrepresented genes. (PDF 555 kb) [file 12859_2019_2683_MOESM7_ESM.pdf]

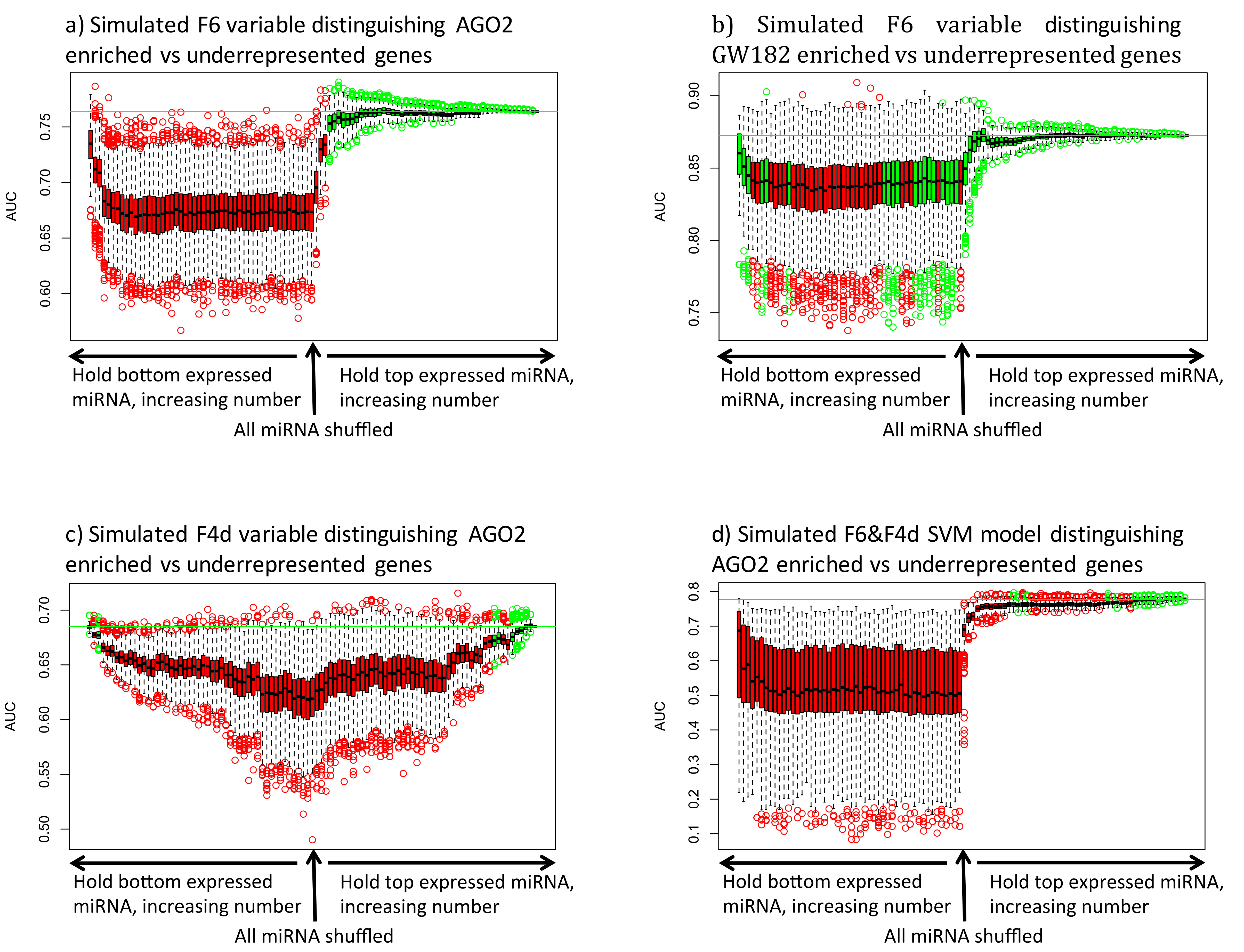
a) Simulated F6 variable distinguishing AGO2 enriched vs underrepresented genes
AUC
0.60
0.65
0.70
0.75
Hold bottom expressed miRNA, increasing number
Hold top expressed miRNA, increasing number
All miRNA shuffled
b) Simulated F6 variable distinguishing GW182 enriched vs underrepresented genes
AUC
0.75
0.80
0.85
0.90
Hold bottom expressed miRNA, increasing number
Hold top expressed miRNA, increasing number
All miRNA shuffled
c) Simulated F4d variable distinguishing AGO2 enriched vs underrepresented genes
AUC
0.50
0.55
0.60
0.65
0.70
Hold bottom expressed miRNA, increasing number
Hold top expressed miRNA, increasing number
All miRNA shuffled
d) Simulated F6&F4d SVM model distinguishing AGO2 enriched vs underrepresented genes
AUC
0.1
0.2
0.3
0.4
0.5
0.6
0.7
0.8
Hold bottom expressed miRNA, increasing number
Hold top expressed miRNA, increasing number
All miRNA shuffled
