# Supplementary material for: RIP-Chip analysis supports different roles for AGO2 and GW182 proteins in recruiting and processing microRNA targets
Source: BMC Bioinformatics. 2019 Apr 18;20(Suppl 4):120. doi: 10.1186/s12859-019-2683-y (PMC6471694; doi:10.1186/s12859-019-2683-y)
Supplement: Supplementary file 9 — Summary of miRNA expression profiles switch between experiment replicas. ROC analysis of F6&F4d SVM model trained with variables calculated with miRNA expression profiles from each of the three anti-AGO2 RIP experiments. SVM models were used to classify the top 1000 and the bottom 1000 genes with respect to the IP/FT mRNA expression ratio, computed for each of the three AGO2 RIP experiments. (PDF 653 kb) [file 12859_2019_2683_MOESM9_ESM.pdf]

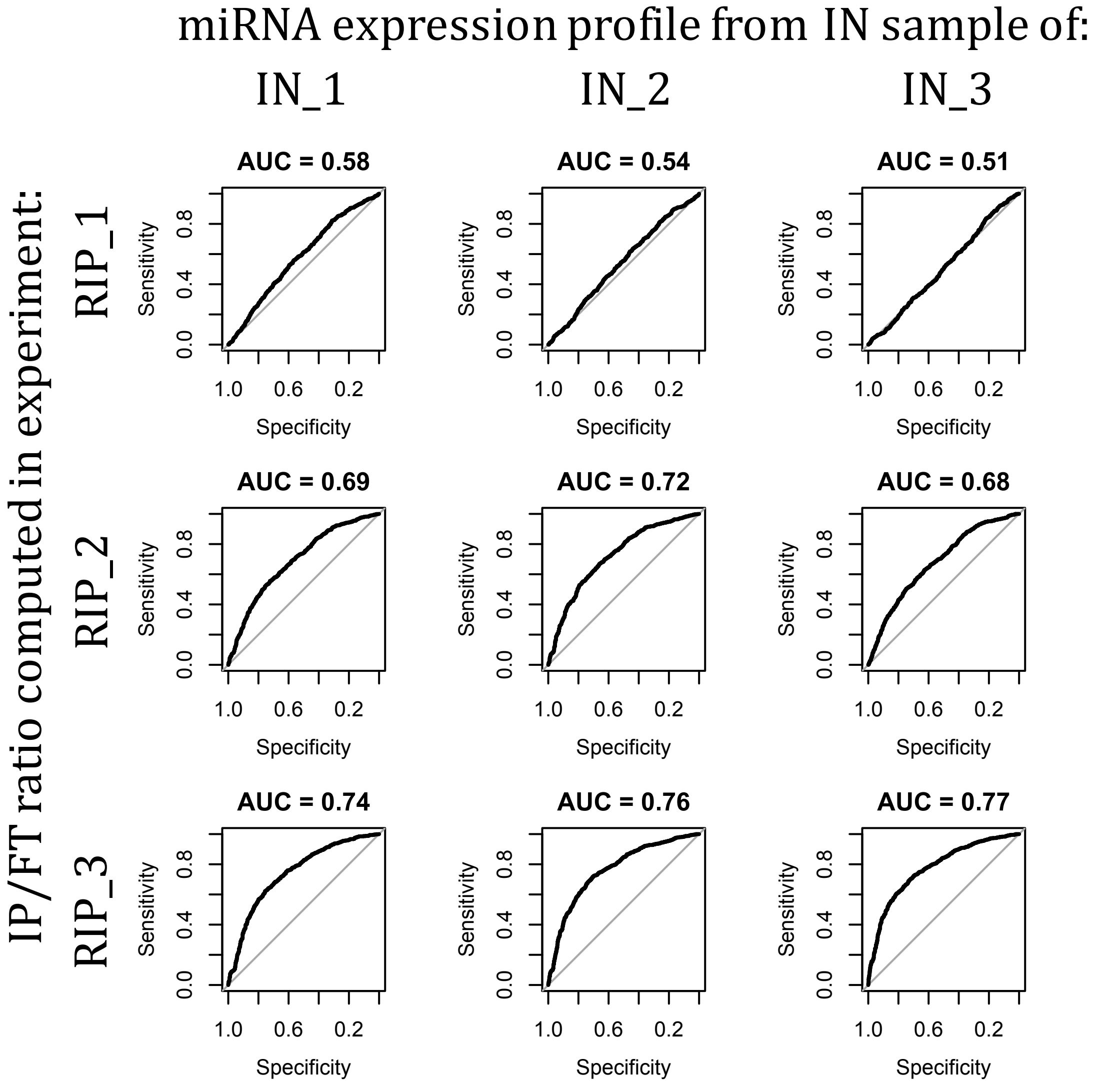
miRNA expression profile from IN sample of:
IN_1
IN_2
IN_3
IP/FT ratio computed in experiment:
RIP_1
RIP_2
RIP_3
AUC = 0.58
AUC = 0.54
AUC = 0.51
AUC = 0.69
AUC = 0.72
AUC = 0.68
AUC = 0.74
AUC = 0.76
AUC = 0.77
Sensitivity
Specificity
0.0
0.4
0.8
1.0
0.6
0.2
